# Cellugyrin (synaptogyrin-2) dependent pathways are used by bacterial cytolethal distending toxin and SARS-CoV-2 virus to gain cell entry

**DOI:** 10.3389/fcimb.2024.1334224

**Published:** 2024-04-18

**Authors:** Kathleen Boesze-Battaglia, Gary H. Cohen, Paul F. Bates, Lisa M. Walker, Ali Zekavat, Bruce J. Shenker

**Affiliations:** ^1^ Department of Basic and Translational Sciences, School of Dental Medicine, University of Pennsylvania, Philadelphia, PA, United States; ^2^ Department of Microbiology, Perelman School of Medicine, University of Pennsylvania, Philadelphia, PA, United States

**Keywords:** cellugyrin, synaptogyrin-2, cytolethal distending toxin, SARS-CoV-2, pathogen, cell entry, synaptic-like microvesicles, Aggregatibacter actinomycetemcomitans

## Abstract

*Aggregatibacter actinomycetemcomitans* cytolethal distending toxin (Cdt) is capable of intoxicating lymphocytes macrophages, mast cells and epithelial cells. Following Cdt binding to cholesterol, in the region of membrane lipid rafts, the CdtB and CdtC subunits are internalized and traffic to intracellular compartments. These events are dependent upon, cellugyrin, a critical component of synaptic like microvesicles (SLMV^Cg+^). Target cells, such as Jurkat cells, rendered unable to express cellugyrin are resistant to Cdt-induced toxicity. Similar to Cdt, SARS-CoV-2 entry into host cells is initiated by binding to cell surface receptors, ACE-2, also associated with cholesterol-rich lipid rafts; this association leads to fusion and/or endocytosis of viral and host cell membranes and intracellular trafficking. The similarity in internalization pathways for both Cdt and SARS-CoV-2 led us to consider the possibility that cellugyrin was a critical component in both processes. Cellugyrin deficient Calu-3 cells (Calu-3^Cg-^) were prepared using Lentiviral particles containing shRNA; these cells were resistant to infection by VSV/SARS-CoV-2-spike pseudotype virus and partially resistant to VSV/VSV-G pseudotype virus. Synthetic peptides representing various regions of the cellugyrin protein were prepared and assessed for their ability to bind to Cdt subunits using surface plasmon resonance. Cdt was capable of binding to a region designated the middle outer loop (MOL) which corresponds to a region extending into the cytoplasmic surface of the SLMV^Cg+^. SARS-CoV-2 spike proteins were assessed for their ability to bind to cellugyrin peptides; SARS-CoV-2 full length spike protein preferentially binds to a region within the SLMV^Cg+^ lumen, designated intraluminal loop 1A. SARS-CoV-2-spike protein domain S1, which contains the receptor binding domains, binds to cellugyrin N-terminus which extends out from the cytoplasmic surface of SLMV. Binding specificity was further analyzed using cellugyrin scrambled peptide mutants. We propose that SLMV^Cg+^ represent a component of a common pathway that facilitates pathogen and/or pathogen-derived toxins to gain host cell entry.

## Introduction

Bacterial protein exotoxins and viruses share the ability to hijack host cell pathways for the purposes of gaining cell entry and trafficking to intracellular target sites to achieve toxicity and, in the case of virus, for efficient replication (Harper et al., 2013). Our recent studies suggest that a unique sorting vesicle, cellugyrin (Cg)-positive synaptic-like microvesicles (SLMVs^Cg+^) mediate entry and subsequent toxicity of bacteria-derived cytolethal distending toxins (Cdts) (Boesze-Battaglia et al., 2016; Boesze-Battaglia et al., 2017; Boesze-Battaglia et al., 2020). Cellugyrin, synaptogyrin-2, is a member of the synaptogyrin tetraspanin protein family (Janz and Sudhof, 1998). Synaptogyrin 1 and 3 are neuronal and are the most abundant proteins in synaptic vesicles; they are critical to vesicle biogenesis, exocytosis and endocytotic recycling as well as neurotransmission (Kedra et al., 1998). Cellugyrin, critical for the biogenesis of SLMVs^Cg+^, is broadly expressed in all tissue (Human Protein Atlas; https://www.proteinatlas.org/ENSG00000108639-SYNGR2/cell), and is considered to be a critical component of SLMVs^Cg+^ (Janz and Sudhof, 1998; Kupriyanova and Kandror, 2000; Belfort and Kandror, 2003; Kioumourtzoglou et al., 2015). SLMVs^Cg+^ are proposed to be early sorting vesicles and contain proteins essential to endocytic processing and are likely a component of the trans golgi network (TGN) (Janz and Sudhof, 1998). Cytoplasmic SLMVs^Cg+^ are positive for Glut4 (Kupriyanova and Kandror, 2000) and phosphatidylinositol 4-kinase type IIα (Xu et al., 2006). Chapel et al. (2013) have also proposed that cellugyrin may function as a lysosomal transporter protein.

Recently, we determined that cellugyrin plays a requisite role in the internalization and endosomal trafficking of Cdts (Boesze-Battaglia et al., 2016; Boesze-Battaglia et al., 2017; Boesze-Battaglia et al., 2020). To date, we have demonstrated that cellugyrin expression in human lymphocytes, macrophages and, more recently, oral keratinocytes is required for toxin entry and subsequent intoxication of these cells (Boesze-Battaglia et al., 2016; Boesze-Battaglia et al., 2017; Boesze-Battaglia et al., 2020; Shenker et al., 2024). Cdts are produced by more than 30 γ- and ϵ-Proteobacteria; indeed, we have shown that cellugyrin is required for at least three Cdts produced by *Aggregatibacter actinomycetemcomitans* (*Aa*Cdt), *Haemophilus ducreyi* (*Hd*Cdt) and *Campylobacter jejuni* (*Cj*Cdt) (Boesze-Battaglia et al., 2017; Boesze-Battaglia et al., 2020; Huang et al., 2021). Cdts are heterotrimeric holotoxins that function as AB_2_ toxins. In this toxin model, the CdtA and CdtC subunits serve as the binding complex (B) and CdtB as the active subunit (A) [reviewed in (Boesze-Battaglia et al., 2016; Scuron et al., 2016)]. Importantly, both CdtB and CdtC are internalized in host target cells such as lymphocytes, macrophages and epithelial cells. The CdtC subunit binds to membrane cholesterol in the context of cholesterol enriched membrane microdomains, or lipid rafts (Boesze-Battaglia et al., 2006; Boesze-Battaglia et al., 2009; Eshraghi et al., 2010; Lin et al., 2011; Zhou et al., 2012; Lai et al., 2013; Boesze-Battaglia et al., 2015; Lai et al., 2015). Within 60 min of exposure to Cdt, cellugyrin translocates from cytoplasm to the plasma membrane in proximity to lipid rafts and associates with CdtB and CdtC. Immunoprecipitation studies demonstrate that cellugyrin, CdtB and CdtC are associated within a molecular complex found both in purified lipid rafts and in the cytoplasm (Boesze-Battaglia et al., 2017; Boesze-Battaglia et al., 2020).

Similar to bacterial exotoxins, viruses must enter host cells to hijack and alter cell function so that the virus can propagate as viral entry into host cells constitutes the initial steps of the infectious life cycle. For enveloped viruses, this involves binding to host cell receptor(s) followed by fusion and/or endocytosis of viral and host cell membranes and intracellular trafficking, respectively (Gillespie et al., 2013; Nelson et al., 2013; Nonnenmacher et al., 2015; Hartenian et al., 2020). These essential functions are dependent upon viral envelope glycoproteins which are utilized by a number of viruses such as Herpes, SARS-CoV and Ebola to achieve entry and infection of host cells (Eisenberg et al., 2012; Ou et al., 2020). The similarities between pathways utilized to intoxicate cells by both bacterial exotoxins and viruses led us to advance the hypothesis that these pathogens utilize a similar mechanism to achieve cell entry. In this context, we proposed that cellugyrin (specifically SLMV^Cg+^) acts as a facilitator for the penetration and internalization of either virus or toxin into target host cells. Indeed, we now demonstrate that cells depleted of cellugyrin expression are not only resistant to Cdt intoxication but also viral infection. Moreover, Cdt, as well as two viral proteins, SARS-CoV-2 spike protein and VSV-G protein are capable of binding to cellugyrin-derived peptides. Collectively, these observations support cellugyrin, or more specifically, SLMV^Cg+^ as a critical host cell pathway utilized by some pathogens such as Cdt producing bacteria and virus such as SARS-CoV-2 to gain cell entry.

## Materials and methods

### Jurkat cells and assessment of Cdt toxicity

The human leukemic T cell line Jurkat was maintained in RPMI 1640 supplemented with 10% FCS, 2 mM glutamine, 10 mM HEPES, 100 U/ml penicillin and 100 g/ml streptomycin as previously described (Shenker et al., 2005). CRISPR/cas9 technology was employed to generate cellugyrin knockout Jurkat cells (Jurkat^Cg-^) using commercially available reagents (Santa Cruz Biotechnology; Santa Cruz, CA) (Boesze-Battaglia et al., 2017). To measure Cdt-induced cell cycle arrest, Jurkat^WT^ (Jurkat^Cg+^) and Jurkat^Cg-^ cells were incubated in the presence of medium or Cdt for 16 hr as previously described (Shenker et al., 2000). Briefly, cells were washed and treated with cold 80% ethanol. After washing, cells were stained with 10 µg/ml propidium iodide containing 1 mg/ml RNase (Sigma Chemical Co; St. Louis, MO) for 30 min. Samples were analyzed on a Becton-Dickinson LSR II flow cytometer (BD Biosciences; San Jose, CA). Propidium iodide fluorescence was excited by an argon laser operating at 488nm and fluorescence measured with a 630/22nm bandpass filter using linear amplification. A minimum of 15,000 events were collected on each sample; cell cycle analysis was performed using Modfit (Verity Software House; Topsham, ME).

### Calu-3 cells, Lentiviral transfection and pseudotype virus infection

Calu-3 cells were obtained from ATCC and maintained in MEM Alpha containing 10% FBS and 1% pennicillin/streptomycin (Gibco). Stable cell lines expressing shRNA against cellugyrin (Calu-3^Cg-^) were prepared with commercially available Lentiviral particles (Santa Cruz Biotech; Dallas, TX); cells were transduced with Lentiviral particles as previously described (Abdul-Sater et al., 2010). Briefly, cells (2.5 x10^4^) were plated and allowed to attach overnight; polybrene (10 μg/mL; Santa Cruz) was added to the cells followed by Lentiviral particles (MOI of 2 - 4; Santa Cruz). After 18 hr, media was replaced with fresh media and cells were allowed to grow for 48 hrs. Calu-3^Cg-^ cells were selected by the addition of puromycin (1 μg/mL; Santa Cruz); surviving cells were cloned by limiting dilution and screened by Western blot for cellugyrin content. Cells were maintened with the addition of puromycin (1 μg/mL) in the culture media.

Pseudotyped VSV-ΔGFP/SARS-CoV2-S (Creative-Diagnostics; Shirley, NY USA) encodes the RNA of a replicon-restricted recombinant Vesicular stomatitis virus (rVSV) in which the glycoprotein (G) gene has been replaced with SARS-CoV2 spike gene. The pseudotyped VSV particles encode GPF together with the VSV nucleocapsid, phosphoprotein and large polymerase subunit in their pVSV-ΔG vector. The VSV- RFP/VSV-G pseudotyped virus (kindly provided by Paul F. Bates) expresses RFP and the VSV-G protein (Anderson et al., 2020). For pseudotype virus challenge, cells (10^5^) were plated and incubated for 5 days in 24 well plates; Calu-3^Cg-^ cells did not receive puromycin for this period. Cells were then challenged with either VSV-ΔRFP/VSV-G or VSV-ΔGFP/SARS-CoV2-S as indicated in serum free media. After 24 hr, cells were harvested and analyzed using a Becton Dickinson LSR II Flow cytometer. RFP and GFP fluorescence were excited with a 488 laser; fluorescence was monitored using 575/26 and 530/30 filters, respectively.

### Surface plasmon resonance

Surface plasmon resonance (SPR) analyses were conducted on a Biacore 3000 (Biacore) at 25°C. The running buffer was PBS containing 0.01 M HEPES (pH 7.4), 3 mM EDTA, and 0.005% surfactant P20 (Biacore). Approximately 2000 response units (RU) of Neutravidin (Thermo Fisher) was coupled to flow cell 2 (Fc2) of a CM5 sensor chip via primary amine coupling according to the manufacturer’s specifications. Fc1 was activated and blocked without the addition of protein. Cellugyrin peptides were constructed with N-terminal biotin (Genscript) and diluted to 500 μg/mL in running buffer and run over both flow cells for 4 min at 5 μL/min and the baseline allowed to stabilize. Once stabilized, Cdt holotoxin, individual Cdt subunits, full length SARS-CoV-2 spike protein [(SARS-CoV-2-S_FL_); ACROBiosystems, Newark,DE] and SARS-CoV-2 S1 spike domain [(SARS-CoV-2-S_1_; Genscript] were diluted to concentrations indicated and binding of each sample was monitored for 5 min, with the wash delay set for an additional 5 min to allow for a smooth dissociation curve. The chip surface was regenerated by injecting brief pulses of 0.2 M sodium carbonate (pH 10) until the response signal returned to baseline. The next cycle was started with the injection of a fresh peptide. SPR data were analyzed with BIAevaluation, version 4.1, which employs global fitting.

### Statistical analysis

Mean ± standard error of the mean was calculated for replicate experiments. Significance was determined using a Student’s t-test with SigmaPlot Software (Systat; San Jose, CA); a P-value of less than 0.05 was considered to be statistically significant.

## Results

### Cdt and SARS-CoV-2 entry and toxicity is dependent upon cellugyrin

Our recent studies demonstrated that entry of Cdt subunits into human lymphocytes, macrophages and epithelial cells is dependent upon the host cell protein, cellugyrin (Boesze-Battaglia et al., 2017; Boesze-Battaglia et al., 2020). Specifically, we have shown that within 60 min of exposure to Cdt, SLMV^Cg+^ translocate to the plasma membrane in proximity to lipid rafts and associates with Cdt subunits CdtB and CdtC. Immunoprecipitation studies also demonstrate that cellugyrin, CdtB and CdtC are associated within a molecular complex found both in purified lipid rafts and in the cytoplasm. Importantly, we have established cell lines with impaired cellugyrin expression for lymphocytes, macrophages and epithelial cells. In all instances, the cellugyrin deficient cells were protected from Cdt toxicity as the active subunit, CdtB was unable to gain intracellular access. An example of this protection is shown in **Supplementary Figure 1** which assesses susceptibility of wildtype Jurkat cells (Jurkat^Cg+^), a human lymphoid cell line and cellugyrin deficient Jurkat cells (Jurkat^Cg-^) to toxin-induced cell cycle arrest. Normally, treatment of Jurkat^Cg+^ cells with Cdt results in cell cycle arrest represented by an increase in the percentage of cells in the G2/M phases of the cell cycle; this increase is demonstrated in **Supplementary Figures 1A, B** as the percentage of G2/M cells increased from 11% in cells incubated with medium alone to 50% in the presence of toxin. In contrast, Jurkat^Cg-^ cells were resistant to these affects (**Supplementary Figures 1C, D**) as the percentage of G2/M cells was essentially the same in control (13.7%) and toxin (12.6%) treated cells. Our collective studies to date suggest that cellugyrin is a critical component of a host cell pathway hijacked by Cdt to achieve entry and intoxicate cells.

**Figure 1:**
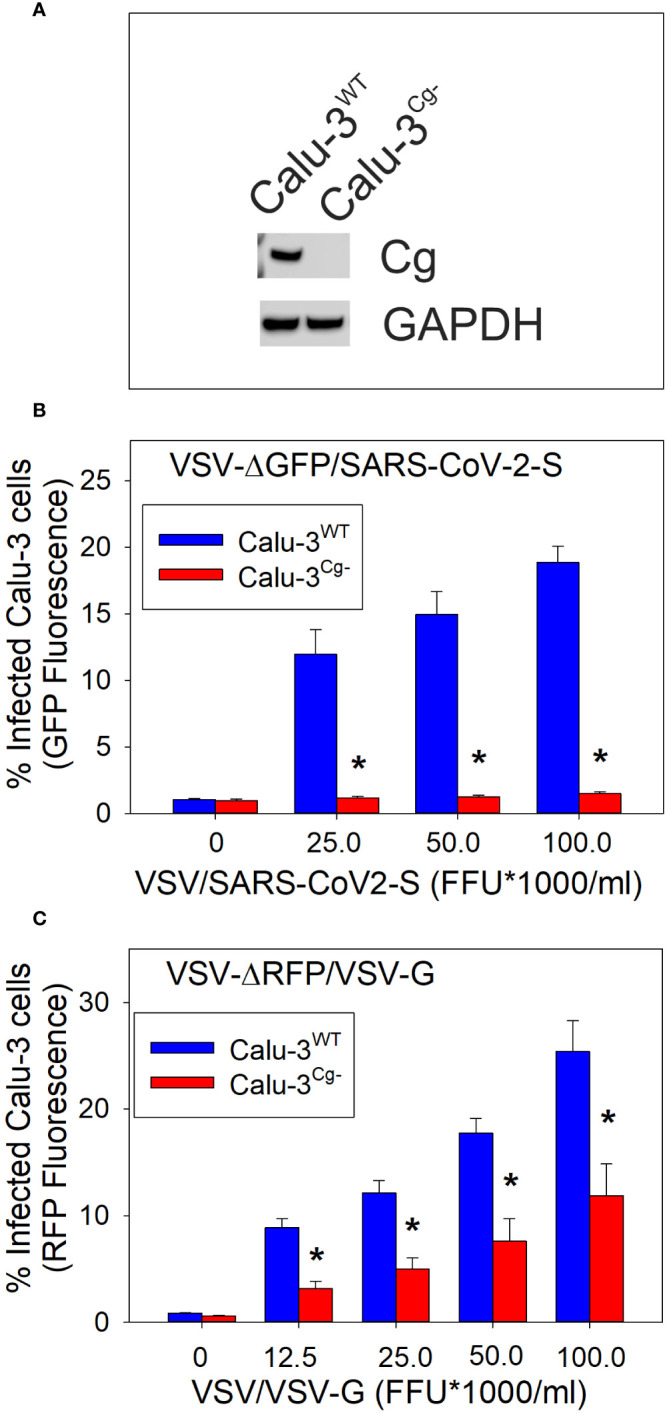
Calu-3 cell susceptibility to SARS-CoV-2 infection is cellugyrin dependent. Calu-3 cells deficient in cellugyrin expression (Calu-3^Cg-)^ were generated using Lentiviral particles as described in Materials and Methods. As shown in panel **(A)** cellugyrin expression was not detectable in Calu-3^Cg-^ cells by Western blot analysis. Correspondingly, the susceptibility of Calu-3^WT^ (Calu-3^Cg+^; blue bars) and Calu-3^Cg-^ (red bars) cells to infection by VSV-ΔGFP/SARS-CoV-2-S pseudotyped virus (panel **B**) or to VSV-RFP/VSV-G pseudotyped virus (panel **C**) were assessed. Cells were challenged with varying FFU for 24 hrs and then analyzed for GFP or RFP fluorescence by flow cytometry. The concentration of FFU is plotted versus the percentage of cells exhibiting increased GFP fluorescence; results are the mean ± SEM of three experiments. *indicates statistical significance (p<0.05).

We now propose that the cellugyrin dependent pathway may also be utilized by viruses to gain cell entry. Here, to demonstrate this relationship we employed the representative pseudotype virus approach for Covid 19. Pseudotype virus was constructed in VSV (lacking VSV-G entry protein) that now contains the SARS-CoV-2 spike protein (VSV-ΔGFP/SARS-CoV-2-S). For comparative purposes we also employed a pseudotype virus which contained the wildtype VSV-G protein (VSV-ΔRFP/VSV-G). The pseudotype viruses contained either the GFP or RFP gene to facilitate detection. Calu-3 cells, a human epithelial lung cancer cell line, were used as target cells. To determine if viral entry is dependent upon cellugyrin, we generated a Calu-3 cellugyrin deficient cell line (Calu-3^Cg-^; **Figure 1A**). Calu-3^WT^ cells were challenged with varying amounts of VSV-ΔGFP/SARS-CoV-2-S pseudotype virus (**Figure 1B**); 24 hr later cell infection was determined by monitoring GFP fluorescence. Calu-3^WT^ infection was dose-dependent and ranged from 12.0 ± 1.8% infected cells in the presence of 25,000 FFU/ml to 18.8 ± 1.2% in the presence of 100,000 FFU/ml virus. In contrast, Calu-3^Cg-^ cells were totally resistant to infection by VSV-ΔGFP/SARS-CoV-2-S pseudotype virus at all doses; the percentage of infected cells never exceeded the value observed in control (untreated) cells, 0.95 ± 0.1%. Calu-3 cells were also challenged with VSV-ΔRFP/VSV-G (0-100,000 FFU/ml) (**Figure 1C**); 24 hr later cells were analyzed for infection by monitoring RFP fluorescence. Calu-3^WT^ cells were infected in a dose-dependent manner; 8.9 ± 0.8% cells were infected when exposed to 12,500 FFU; the percentage increased with each dose of virus to a maximum of 25.4 ± 2.9% in the presence of 100,000 FFU/ml, the highest viral concentration employed. In contrast, Calu-3^Cg-^ cells exhibited significantly reduced infection when challenged with the same doses of pseudotype virus (12,500 to 100,000): 3.1 ± 0.7%, 5.0 ± 1.0%, 7.6 ± 1.4% and 11.9 ± 2.9%.

### Cdt binds to cellugyrin peptides

**Figure 2:**
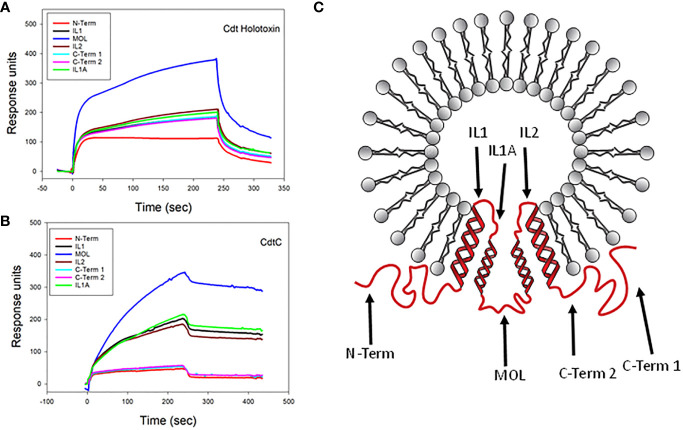
SPR analysis of Cdt binding to cellugyrin peptides. The interaction of Cdt holotoxin **(A)** and CdtC **(B)** with the seven cellugyrin peptides were analyzed by SPR. Representative sensorgrams for the interaction of Cdt holotoxin (1.0 µM) and CdtC (4.0 µM) with cellugyrin peptides are shown and plotted as response units versus time. Sensorgrams are representative of at least three repetitive experiments. **(C)** shows a topographical model of cellugyrin within SLMV^Cg+^. Cellugyrin is shown in red along with the 7 peptides that were designed (black arrows): N-terminus (N-Term), C-terminus (C-Term) 1 and C-Term 2 and middle loop (MOL) face the cytoplasmic surface of the vesicles. The intraluminal loops (IL) are designated IL, IL1A and IL2. Additionally, the four transmembrane regions (TM1-4) are shown. Adapted from Low et al. (2012).

Previously, we demonstrated that Cdt subunits co-localize with cellugyrin in the vicinity of cholesterol-rich microdomains in both human lymphocytes and macrophages and, as noted above, form molecular complexes detected by immunoprecipitation (Boesze-Battaglia et al., 2017; Boesze-Battaglia et al., 2020). Prior to our current studies, we had not demonstrated direct binding between the Cdt subunits and cellugyrin. We now assessed the ability of Cdt holotoxin and individual subunits to bind directly to synthetic cellugyrin peptides; these analyses not only allow us to identify regions of cellugyrin association but also to predict the binding site location within the context of SLMV^Cg+^. For this purpose, peptides were designed based upon cellugyrin sequence and topography within SLMV^Cg+^; a topology model for cellugyrin within SLMV^Cg+^ is presented in **Figure 2** (Stenius et al., 1995; Low et al., 2012). In this rendition, the N-terminus and C-terminus extend into the cytoplasm; the middle outer loop (MOL) links transmembrane (TM) regions TM2 with TM3 and is exposed to the cytoplasmic surface of SLMV^Cg+^ as well. Additionally, there are two loops within the lumen of SLMV^Cg+^ designated inner loop (IL) 1 (IL1) which links TM1 and TM2 and IL2 which links TM3 and TM4.

Based on these predictions, seven peptides, corresponding to each of the inner and outer cellugyrin loop domains as well as both the N– and C-terminus, were designed and synthesized as described in Materials and Methods (**Table 1**; **Figure 2**). Due to length of the IL1 loop and C-terminus, two peptides covering this region were designed and designated IL1, IL1A, C-Term 1 and C-Term 2. SPR (Biacore) was employed to assess Cdt subunit and holotoxin binding to the immobilized cellugyrin peptides. Analysis of the binding of Cdt holotoxin, containing all three subunits indicated maximum binding to the MOL peptide (**Figure 2A**). While the Cdt holotoxin exhibited binding to all peptides, preferential binding (maximum RU 380) was observed with the MOL peptide. Similarly, the Cdt binding subunit, CdtC, also exhibited the highest RU (340) with the MOL peptide (**Figure 2B**); moderate interactions were observed with IL1, IL1A and IL2. No interactions were observed with the N-Term or with either of the two C-Term peptides. Also, we did not detect interactions with any of the cellugyrin peptides when the CdtB subunit and CdtA subunit were assessed individually (data not shown).

**Table 1:** Cellugyrin peptides.

Name	Sequence
N-Term	^2^ESGAYGAAKAGGSF^15^
Inner loop 1 (IL1)	^45^GEGYSNAHESKQMYC^59^
Inner loop 1A (ILA)	^57^MYCVFNRNEDACRYG^71^
Middle outer loop (MOL)	^100^DRKYLVIGDLLFSA^113^
Inner loop 2 (IL2)	^125^LTNQWAVTNPKDVLVGAD^143^
C-Term 1	^173^GVDDFIQNYVDPTPDP^188^
C-Term 2	^211^NAETTEGYQPPPVY^224^

**Figure 3:**
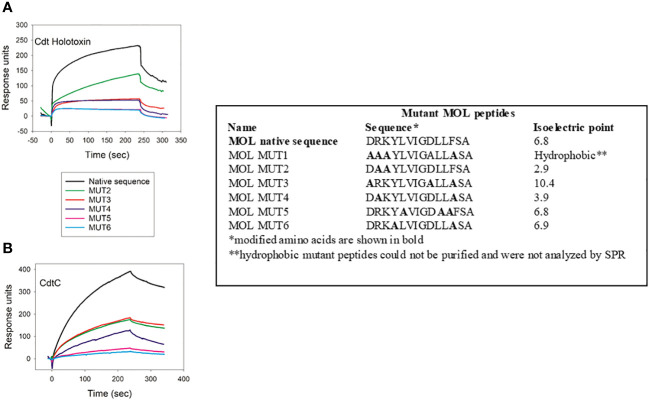
Charged amino acid residues in cellugyrin MOL are necessary for Cdt binding. The interaction of 1 µM Cdt holotoxin **(A)** and 4.0 µM CdtC **(B)** with MOL mutant peptides was analyzed by SPR. Sequence of mutated peptides and change in pI are shown. Representative sensorgrams for the interaction of Cdt proteins with wildtype and mutant MOL peptides are shown and plotted as response units versus time. Sensorgrams are representative of at least two repetitive experiments.

We next tested criteria for peptide binding and specificity with the MOL region (**Figure 3**). Specifically, we employed MOL mutant peptides in which isoelectric point was altered and aromatic resides were replaced with alanine, individually and in combination, as illustrated in **Figure 3**. Cdt holotoxin bound to cellugyrin MOL MUT2; in this mutant basic residues were replaced with alanine leading to a marked reduction of the isoelectric point from 6.8 to 2.9. Binding however, was only 50% of the maximum RU when compared to the native peptide (**Figure 3A**). Binding of the holotoxin was abolished with all other peptide combinations (MOL MUT3-MUT6). When CdtC binding was assessed using the same panel of cellugyrin MOL mutants, binding was abolished when aspartic acid (D) residues were replaced by alanine regardless of whether phenylalanine (F) was also replaced (MOL MUT2 and MOL MUT3; **Figure 3B**). Binding to MOL MUT5 in which the phenylalanine was retained, while lower than native peptide was measurable, inferring the essentiality of aspartic acid. Collectively, these series of mutant MOL studies suggest that the interaction between Cdt holotoxin as well as the Cdt C subunit may rely on charged amino acid residues. Going forward we will assess the contribution of the individual charged residues, both acidic and basic, to optimal Cdt-cellugyrin association.

### SARS-CoV-2 binds to cellugyrin peptides

**Figure 4:**
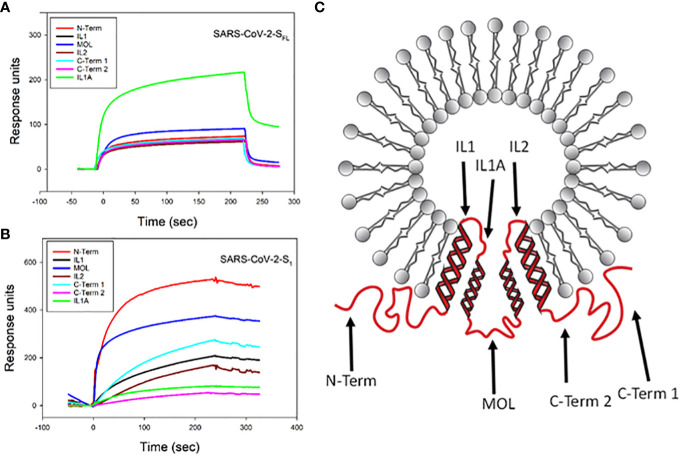
SPR analysis of viral protein binding to cellugyrin peptides. The interaction of 2.5 µM SARS-CoV-2-S_FL_ **(A)** and 2.5 µM SARS-CoV-2-S_1_ **(B)** with cellugyrin peptides were analyzed by SPR. Sensorgrams for the interaction of viral proteins with cellugyrin peptides are shown and plotted as response units versus time. Sensorgrams are representative of at least three repetitive experiments. **(C)** shows a topographical model of cellugyrin within SLMVCg+. Cellugyrin is shown in red along with the 7 peptides that were designed (blue arrows): N-terminus (N-Term), C-terminus (C-Term) 1 and C-Term 2 and middle loop (MOL) face the cytoplasmic surface of the vesicles. The intraluminal loops (IL) 1, IL1A and IL2. Additionally, the four transmembrane regions (TM1-4) are shown. Adapted from Low et al. (2012).

Next, we used a similar approach to assess the ability of viral proteins to bind to cellugyrin peptides; these included full length SARS-CoV-2 spike protein (SARS-CoV-2-S_FL_), SARS-CoV-2 spike domain 1 containing the RBD (SARS-CoV-2-S_1_) and VSV-G. SARS-CoV-2-S_FL_ exhibited binding to IL1A with a maximum RU of 220 (**Figure 4A**); low binding was observed with the other peptides exhibiting a maximum RU range of 55 to 95. It should be noted that IL1 and IL1A collectively represent the inner loop 1 region with minimal overlap. In contrast, SARS-CoV2-S_1_ preferentially bound to the N-Term peptide with a maximum RU of 530 (**Figure 4B**); this protein also exhibited binding to the other peptides with lower RUs ranging from 50 to 380. VSV-G exhibited maximal binding to the MOL peptide with a peak RU of 140 (**Supplementary Figure 2A**).

**Figure 5:**
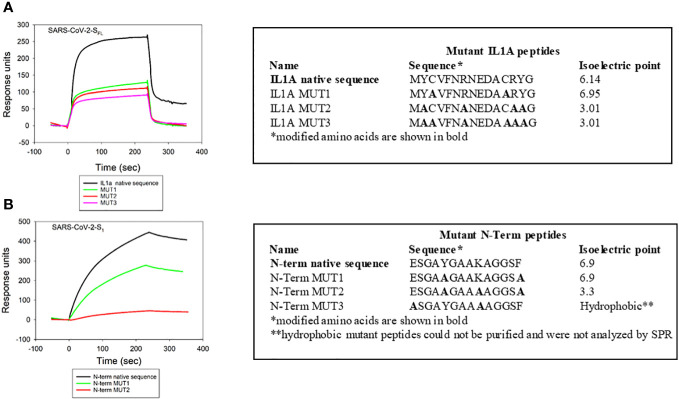
Viral protein binding to cellugyrin is dependent on charged amino acid residues. The interaction of 2.5 µM SARS-CoV-2-S_FL_ with IL1A mutants **(A)** and 2.5 µM SARS-CoV-2-S_1_ with N-Term mutants **(B)** was analyzed by SPR. Mutation sequences and how they deviate from native protein sequence is shown. Representative sensorgrams of at least two repetitive experiments are shown for the interaction of viral proteins with wildtype and mutant IL1A and N-Term peptides are shown and plotted as response units versus time.

Similar to our approach to understanding the specificity of Cdt binding to cellugyrin peptides, we also generated mutant peptides corresponding to regions of interaction between cellugyrin and SARS-CoV-2-S_FL_ (IL1A) and SARS-CoV-2-S_1_ (N-Term) (**Figure 5**). IL1A mutant peptides represent changes in charged and cysteine residues (**Figure 5A**). In IL1A MUT1, cysteine was replaced with alanine, in IL1A MUT2 and IL1A MUT3, hydrophobicity and charge were decreased; in IL1A MUT3 we also replaced cysteine with alanine. SARS-CoV-2-S_FL_ binding to IL1A MUT1 and IL1A MUT2 was reduced by more than 50% relative to the wildtype peptide (**Figure 5A**). In IL1A MUT3, both the cysteine residues and tyrosine residues were replaced with alanine leading to a 70% decrease in SARS-CoV-2-S_FL_ binding. Collectively, these studies suggest that SARS-CoV-2-S_FL_ relies on a combination of cysteine and aromatic residues for maximal binding The potential importance of a disulfide bridge will be considered in future studies. The N-terminal mutant peptides were assessed and modified as follows (**Figure 5B**): N-Term MUT1 hydrophobicity was reduced and N-Term MUT2 isoelectric point was lowered (**Figure 3**). SARS-CoV-2-S_1_ exhibited a 25% reduction in binding with N-Term MUT1 while loss of both hydrophobicity and basic charge (lysine) abolished binding in N-Term MUT2 (**Figure 5B**).

VSV-G binding to MOL mutants was assessed using the same mutant peptides as employed above for Cdt holotoxin and CdtC (**Figure 3**). Similar to Cdt holotoxin and CdtC, VSV-G binding was abolished with MOL MUT5 and MOL MUT6 (**Supplementary Figure 2B**), The other MOL mutants exhibited 30-50% reduced binding based on maximal RU.

**Figure 6:**
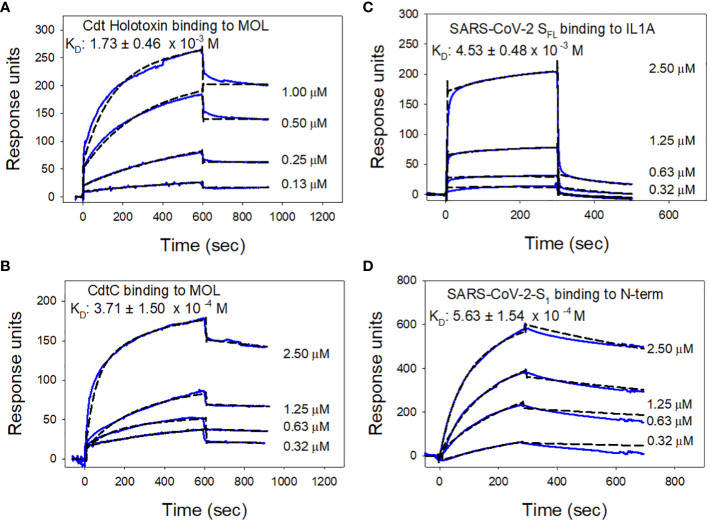
Analysis of toxin and viral protein binding affinities to cellugyrin peptides. Representative sensorgrams for the interaction of various concentrations of Cdt holotoxin **(A)** and CdtC **(B)** to MOL is shown. Sensorgrams for the interaction of various concentrations of SARS-CoV-2-S_FL_ with IL1A **(C)** and SARS-CoV-2-S_1_ with N-Term **(D)** are shown. Experimental line (solid) is shown along with the model fit (broken line). K_D_ values were calculated from these data and the mean ± SEM of three experiments are shown in each panel; sensorgrams are representative of at least three repetitive experiments.

Additional SPR analyses were conducted to determine binding affinities for each of the pathogen proteins with the cellugyrin peptide to which they exhibited preferential reactivity as described above. **Figure 6** shows representative series of overlay sensorgram responses for multiple concentrations of Cdt holotoxin (**Figure 6A**) and CdtC subunit (**Figure 6B**) binding to MOL. Both actual data (solid lines) and modeled data (dashed lines) are plotted. K_D_ values were calculated for Cdt holotoxin and CdtC binding to the MOL as 1.73 ± 0.46 x 10^-3^ M and 3.7 ± 1.5 x 10^-4^ M, respectively. Similarly, sensorgram overlays for varying concentrations of each of the viral proteins are shown for SARS-CoV-2-S_FL_ binding to IL1A (**Figure 6C**) and SARS-CoV-2-S_1_ binding to N-term (**Figure 6D**); the calculated K_D_ values are 4.53 ± 0.48 x 10^-3^ M, 5.63 ± 1.54 x 10^-4^ M, respectively.

## Discussion

Cdt is able to intoxicate multiple host cell types by virtue of its exploitation of a ubiquitous cell receptor, cholesterol, and its molecular mode of action which disrupts phosphatidylinositol-3 kinase (PI-3K) signaling by enzymatically depleting cells of PIP3 (Boesze-Battaglia et al., 2006; Shenker et al., 2007; Boesze-Battaglia et al., 2009; Shenker et al., 2010; Shenker et al., 2014; Boesze-Battaglia et al., 2015). Moreover, following CdtC binding to cholesterol, internalization and intracellular trafficking of CdtB involves utilization of the host protein, cellugyrin (Boesze-Battaglia et al., 2017; Boesze-Battaglia et al., 2020). In the absence of cellugyrin, CdtB does not enter cells and is unable to induce toxicity. Moreover, we have previously demonstrated that Cdts derived from *Haemophilus ducreyi* and *Campylobacter jejuni* are also dependent upon cellugyrin for entry and toxicity (Huang et al., 2021). In this study we demonstrate for the first time that both Cdt holotoxin and the toxin’s CdtC cholesterol binding subunit directly interact with cellugyrin. Specifically, these toxin entities bind to the peptide containing a sequence that corresponds to a region representing the MOL; it is noteworthy that this region resides on the cytoplasmic surface of SLMV^Cg+^ (see **Figure 2C**). Moreover, we have previously shown that SLMV^Cg+^ accumulate within the cholesterol rich lipid membrane rafts following exposure to Cdt. The relative affinity for the MOL peptide was determined to be higher for the CdtC subnunit than that observed for the holotoxin (K_D_ values were 3.71 ± 1.50 x 10^-4^ M and 1.73 ± 0.46 x 10^-3^ M, respectively). We did not detect significant binding of the CdtA subunit and the active Cdt subunit, CdtB, to cellugyrin peptides (data not shown). However, it should be noted that CdtB entry is not only dependent upon CdtC, but both subunits enter cells and initially remain part of a multi-protein-cellugyrin positive complex detectable by immunoprecipitation (Boesze-Battaglia et al., 2017). Moreover, it is possible that CdtB binding to cellugyrin may involve cellugyrin sequences not represented within the pool of peptides employed in the current study. It should also be noted that the inability of the CdtA subunit, also a cell surface binding subunit, to interact with cellugyrin is consistent with the observation that this subunit does not enter host cells (Boesze-Battaglia et al., 2017). In addition to our previous studies demonstrating that CdtB requires cellugyrin for internalization and intoxication of host cells, we now demonstrate and identify regions of direct interaction between toxin and the host cell protein cellugyrin.

Viral entry into host cells follows a similar pattern to that described above for bacterial exotoxins such as Cdts: recognition and binding to host cell receptors, translocation across the plasma membrane and hijacking of intracellular transport mechanisms (Eisenberg et al., 2012; Glebov, 2020; Hartenian et al., 2020; Scudellari, 2021). In this regard, SARS-CoV-2 expresses a spike protein (S) with three receptor-binding heads (S1) that sit on top of a fusion stalk (S2). S1 contains the receptor binding domain (RBD) that specifically recognizes angiotensin-converting enzyme 2 (ACE2) as it’s host cell receptor. Following RBD binding to ACE2, viral and host cell membrane fusion takes place; this process involves host cell protease-dependent modification of S2 (which is able to function as a fusogen) following its dissociation from S1 (Ou et al., 2020; Shang et al., 2020; Wan et al., 2020). Following fusion, endosome formation and maturation leads to release of viral RNA and hijacking of the protein synthetic machinery of the cell to synthesize viral proteins that ultimately assemble into more copies of the virus. Another similarity worth noting between SARS-CoV-2 (and SARS-CoV) interaction with host cells and that observed for Cdt is that cell entry involves interactions associated with membrane lipid rafts (Li et al., 2007; Lu et al., 2008; Fecchi et al., 2020) as ACE-2 has been reported to associate within these cholesterol-rich membrane regions. The requirement for the integrity of these regions was further shown by the observation that cholesterol depletion protects cells from viral entry; similar observations have been made for Cdt (Boesze-Battaglia et al., 2009; Eshraghi et al., 2010; Lin et al., 2011; Lai et al., 2013; Boesze-Battaglia et al., 2015; Boesze-Battaglia et al., 2016). These similarities to Cdt entry led us to consider the possibility that SARS-CoV-2 might also interact with, and be dependent upon, cellugyrin.

**Figure 7:**
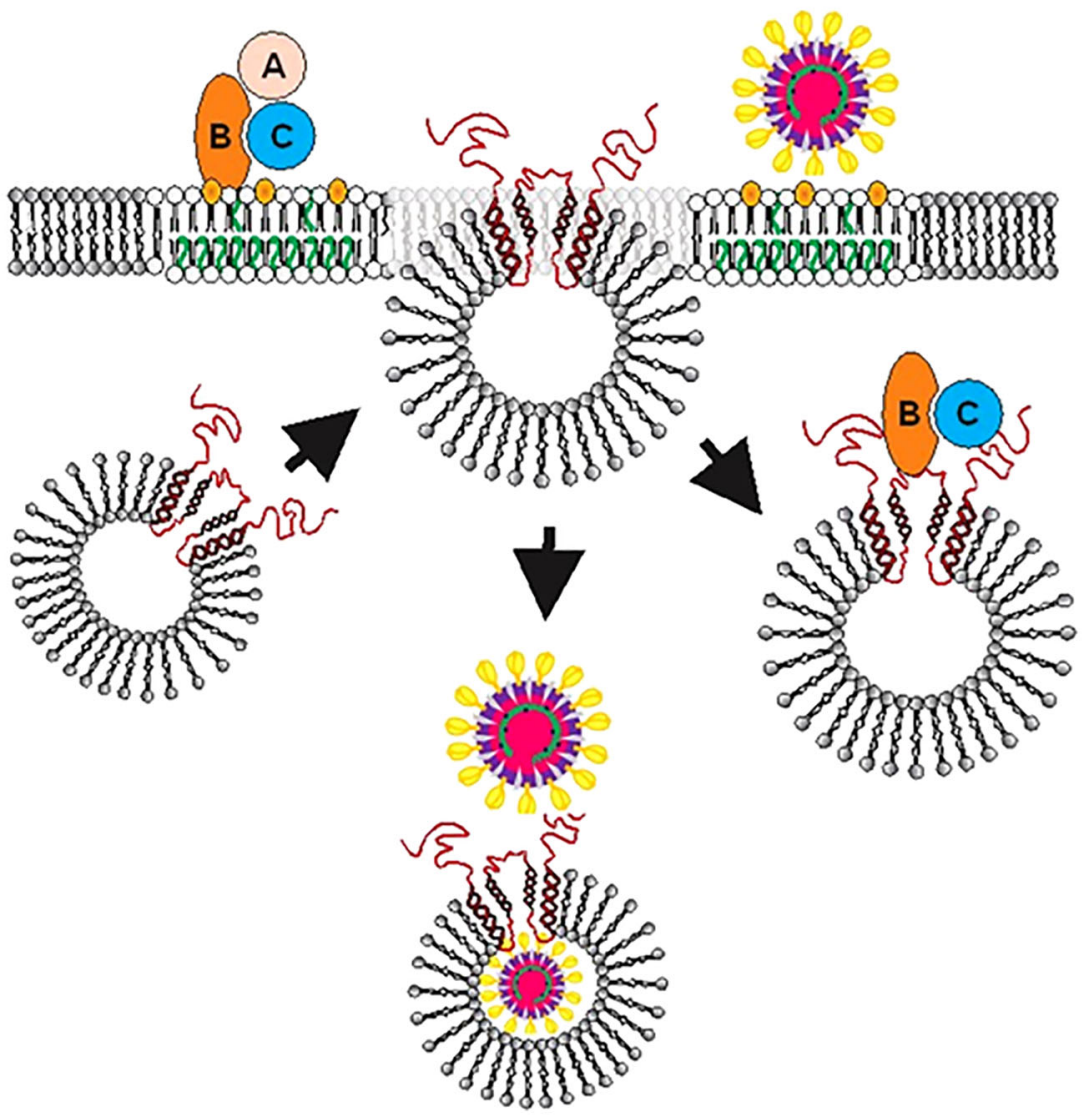
Proposed model for the role of SLMV^Cg+^ in facilitating Cdt or viral entry into host cells and intracellular trafficking. After binding of toxin (Top left) or virus (Top right) to lipid raft associated receptors, SLMV^Cg+^ translocate from cytoplasm to plasma membrane allowing cellugyrin (red) to be exposed to the extracellular surface and associate with pathogen binding proteins. For viruses such as SARS-CoV-2 we further propose that a second step (also occurring in close proximity to the plasma membrane) involves fusion events that ultimately lead to viral translocation to the SLMV^Cg+^ lumen. Orange represents cholesterol and green unsaturated fatty acids within the host cell membrane.

Clearly, our observations demonstrate that cellugyrin is critical for SARS-CoV-2 infection as we demonstrated that cells with impaired expression of this protein (Calu-3^Cg-^ cells) were resistant to infection by VSV-ΔGFP/SARS-CoV-2-S pseudotype virus. SARS-CoV-2-S_FL_ binds to the cellugyrin peptide containing an amino acid sequence corresponding to IL1A with a similar affinity as the Cdt proteins (K_D_ 4.53 ± 0.48 x 10^-3^ M). This region is present within the vesicle lumen (**Figure 4C**); access to this region of the tetraspanin protein is likely consistent with fusion processes that have been proposed for the early stages of viral entry. Indeed, the involvement of fusion with SLMV^Cg+^ is supported by the observation that these vesicles contain the v-SNARE fusion protein (Kupriyanova and Kandror, 2000). SLMV^Cg+^ have been shown to traffic to the plasma membrane under basal conditions; this association and likely subsequent fusion is mediated through a plasma membrane associated t-SNARE (Kioumourtzoglou et al., 2015). Moreover, SLMV^Cg+^ fusion with the plasma membrane has been demonstrated in response to insulin signaling to deliver GLUT4 (Kupriyanova and Kandror, 2000; Kupriyanova et al., 2002). Interestingly, the spike S1 domain preferentially binds to the N-terminus which extends from the SLMV^Cg+^ surface into the cytoplasm (K_D_ 5.63 ± 1.540 x 10^-4^ M). Collectively, these observations allow for the possibility of two-stage interaction between SARS-CoV-2 spike protein and cellugyrin. For example, initial viral-cellugyrin binding may involve the S1 domain of the spike protein binding to the N-terminus of cellugyrin; this may be followeded by fusion and/or reorganization of the SLMV^Cg+^ allowing for interaction with the IL1A region (**Figure 7**). Clearly, future studies will focus on advancing our understanding of the involvement of these regions in SARS-CoV-2 infection.

We also assessed the VSV-G protein for its ability to interact with cellugyrin. The VSV-G protein, similar to the SARS-CoV-2 spike protein, is required for binding to host cell receptors and subsequent internalization and cell infection (Oksayan et al., 2012; Regan and Whittaker, 2013). Interestingly, VSV-G, like Cdt holotoxin and CdtC, preferentially binds to the MOL. In contrast to our observations with VSV-ΔGFP/SARS-CoV-2, Calu-3^Cg-^ cells were partially protected from infection with the VSV-ΔRFP/VSV-G pseudotype virus. Failure to totally block VSV-ΔRFP/VSV-G pseudotype viral entry likely reflects VSV’s known ability to utilize multiple pathways to gain host cell entry (Oksayan et al., 2012; Regan and Whittaker, 2013).

There is additional evidence in support of cellugyrin, or more specifically SLMV^Cg+^, serving as a critical host cell protein utilized by viruses for the purposes of intracellular trafficking and infection. For example, Sun et al. (2016) reported on the role of cellugyrin in the infection of mammalian cells by Bunyavirus which is responsible for severe fever with thrombocytopenia syndrome virus (SFTSV). Cellugyrin was shown to interact with nonstructural viral proteins and further, these interactions led to the transport of these proteins into inclusion bodies that were “reconstructed from lipid droplets” during viral infection. The importance of this translocation was demonstrated by silencing cellugyrin expression which resulted in reduced inclusion body formation and viral titers. More recently, Walker et al. (2018) reported on Porcine circovirus 2 (PCV2) which is responsible for a group of diseases known as PCV2 Associated Disease. PCV2 disease exhibits variation in incidence and severity of disease; investigators proposed that this variability is due to missense mutation in the SYNGR2 gene (cellugyrin) resulting in altered viremia. Experimental silencing of SYNGR expression was found to significantly reduce PCV2 titers in infected PK15 cells. It was proposed that cellugyrin mutation(s) affect virus incorporation into vesicular membranes and thereby alter vesicle formation; the altered vesicles fail to transport PCV2 resulting in impaired viral replication.

The observations reported in this study led us to propose that similar to exotoxins such as Cdt, viruses such as SARS-CoV-2 hijack the cellugyrin-dependent pathway to access intracellular compartments within host target cells. Noteworthy, in a recent study on the upper airway host transcriptional response of patients with COVID-19 to other viral lung infections SYNGR2 was found to be upregulated >1.035-fold and 1.35-fold when compared with nonviral respiratory disorders (Mick et al., 2020). In light of our current observations with the bacterial exotoxin, Cdt, and SARS-CoV-2, we now propose that SLMV^Cg+^, and cellugyrin in particular, represent a universal facilitator for an array of pathogens, or their virulence factors, to achieve host cell entry. Moreover, a developing theme in these interactions is the involvement of cholesterol-rich microdomains as a region in which pathogen, host cell receptor and cellugyrin likely come together to initiate the internalization process(es). In conclusion, our study provides evidence that viruses and exotoxins alike, utilize a *common mechanism* to achieve cell entry (**Figure 7**). We propose that hijacking of this SLMV^Cg+^ pathway is a requisite for both bacterial and viral infection which leads to altered host cell function which, in turn, contributes to disease pathogenesis. Clearly, modulation of cellugyrin expression or interference with pathogen-cellugyrin interaction represent novel, non-anti-microbial, approaches to prevent and/or treat a range of viral and bacterial-induced disorders.

## Data availability statement

The raw data supporting the conclusions of this article will be made available by the authors, without undue reservation.

## Author contributions

KB-B: Conceptualization, Data curation, Formal analysis, Funding acquisition, Investigation, Methodology, Project administration, Resources, Supervision, Validation, Visualization, Writing – original draft, Writing – review & editing. GC: Conceptualization, Formal analysis, Methodology, Writing – review & editing. PB: Methodology, Resources, Writing – review & editing. LW: Data curation, Formal analysis, Investigation, Methodology, Writing – review & editing. AZ: Data curation, Formal analysis, Investigation, Methodology, Writing – review & editing. BS: Conceptualization, Data curation, Formal analysis, Funding acquisition, Investigation, Methodology, Project administration, Resources, Supervision, Validation, Visualization, Writing – original draft, Writing – review & editing.
