# Supplementary material for: Cellugyrin (synaptogyrin-2) dependent pathways are used by bacterial cytolethal distending toxin and SARS-CoV-2 virus to gain cell entry
Source: Front Cell Infect Microbiol. 2024 Apr 18;14:1334224. doi: 10.3389/fcimb.2024.1334224 (PMC11063343; doi:10.3389/fcimb.2024.1334224)
Supplement: Supplementary file 1 [file DataSheet_1.pdf]

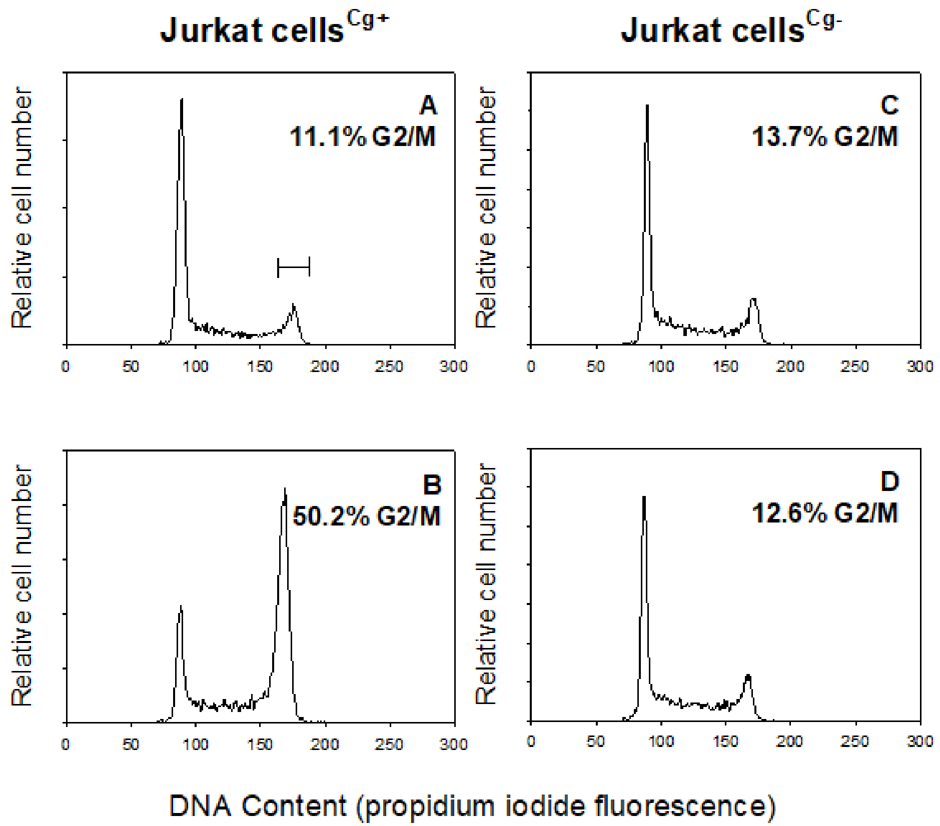

Supplementary Figure 1. Cellugyrin expression is required for *Aa*Cdt-induced cell cycle arrest. Jurkat wildtype cells (Jurkat^{Cg+}) and CRISPR/Cas9 modified Jurkat cells which do not express cellugyrin (Jurkat^{Cg-}) were exposed to medium alone (panels A and C) or to 10 pg/ml Cdt (panels B and D) for 24 hr. Cells were then assessed for cell cycle distribution by monitoring propidium iodide fluorescence by flow cytometry; bar in panel A shows gating for G2/M cells used for all data presented in panels A-D. Cdt-induced cell cycle arrest in Jurkat^{Cg+} cells is demonstrated by an increase in the percentage of cells in the G2/M phase; in contrast, Jurkat^{Cg-} were resistant to Cdt as the percentage of cells in G2/M in did not change from that observed in control cells. Results are representative of three experiments; numbers in each panel represent the percentage of G2/M cells.

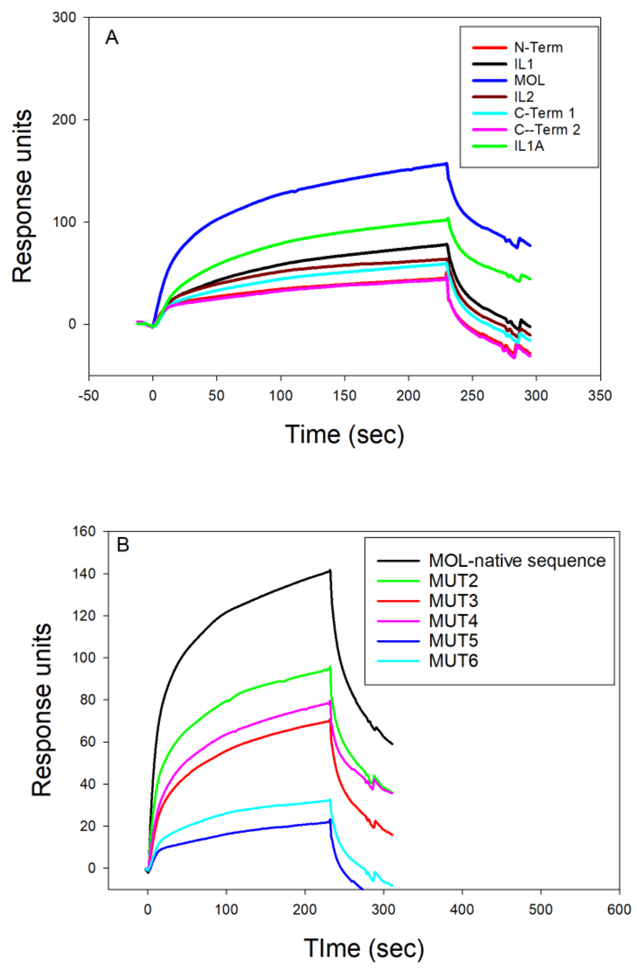

Supplementary Figure 2. SPR analysis of VSV-G protein binding to cellugyrin peptides (A) and MOL mutant peptides (B). The binding of VSV-G (4 µM) to cellugyrin peptides and MOL mutant peptides were analyzed by SPR. Representative sensorgrams (of three experiment) for the interaction of the VSV-G-protein with peptides are shown and plotted as response units versus time.
